# Psoriasis Beyond the Skin: A Disease With Cardiovascular Risk

**DOI:** 10.7759/cureus.88464

**Published:** 2025-07-21

**Authors:** Maria Cristofori, José C González-Rodríguez, Emmanuel E Cortés-Marín, Adipp Sallón, Jairo Sandoval

**Affiliations:** 1 General Practice, Universidad de Ciencias Médicas (UCIMED), San José, CRI; 2 Internal Medicine, Universidad de Costa Rica, San José, CRI; 3 Emergency Department, Hospital México, San Jose, CRI; 4 Intensive Care Unit, Hospital México, San José, CRI

**Keywords:** atherosclerosis, autoimmunity, cardiovascular disease, cytokines, psoriasis, tumor necrosis factor-alpha

## Abstract

Psoriasis is a chronic immune-mediated skin disease that is increasingly understood as a systemic inflammatory condition with implications that extend far beyond the skin. Among its most serious associations is an elevated risk of cardiovascular disease, which has emerged as a leading cause of morbidity and mortality in affected patients. The persistent immune activation characteristic of psoriasis, driven by cytokines such as tumor necrosis factor α (TNFα), interleukin (IL)-17, and IL-23, contributes to endothelial dysfunction, oxidative stress, and atherogenesis. This shared pathophysiology helps explain the increased prevalence of coronary artery calcification, impaired microvascular function, and early-onset myocardial infarction observed in this population. Traditional risk assessment tools often fail to capture the actual cardiovascular burden in patients with moderate to severe disease. Evidence suggests that biologic therapies targeting key inflammatory pathways not only improve dermatologic outcomes but may also mitigate vascular risk, offering systemic benefits that extend beyond skin clearance. Recognizing psoriasis as a multisystem disorder reinforces the need for a more integrated approach to risk assessment and long-term management.

## Introduction and background

Psoriasis is a chronic inflammatory skin disease that affects approximately 60 million people worldwide, with a global prevalence of 3%. This condition extends beyond dermatological concerns, as it is strongly linked to several systemic comorbidities, particularly cardiovascular disease, which significantly impacts morbidity and mortality [1-3]. Sustained inflammatory signaling activates pathways involving tumor necrosis factor α (TNFα), interleukin (IL)-17, and IL-23, leading to endothelial dysfunction, oxidative stress, and the advancement of atherosclerosis [4]. These changes are reflected in biomarkers such as elevated C-reactive protein and increased levels of vascular endothelial growth factor (VEGF), as well as imaging findings, including coronary artery calcification and microvascular abnormalities, all of which support the concept of a "psoriatic march" toward vascular involvement [4-6]. In response, targeted therapies against TNF-α, IL-17, and IL-23 have demonstrated the potential to partially reverse these systemic alterations [5,6]. Given the intricate interplay between psoriasis and vascular inflammation, a multidisciplinary approach involving dermatology, internal medicine, and cardiology is essential. This strategy facilitates a comprehensive assessment and management of cardiovascular risk, complementing skin disease control with therapeutic interventions aimed at enhancing vascular health. This review aims to examine the latest evidence on the shared pathogenic mechanisms between psoriasis and cardiovascular disease, as well as the impact of available treatments, particularly biologics, thereby providing a multidimensional clinical approach that enhances outcomes for patients with psoriasis.

## Review

Psoriasis is far more than a skin condition. It's an immune-mediated disease driven by T-cell hyperactivity, which causes keratinocytes to proliferate and differentiate abnormally. This process ultimately compromises the skin barrier's integrity. But the dysfunction doesn't stop at the skin; it is also linked to various systemic comorbidities, including rheumatologic, cardiovascular, and psychiatric disorders [1,7]. In fact, cardiovascular diseases have become the leading cause of morbidity and mortality for individuals with psoriasis. This has established psoriasis as an independent risk factor for cardiovascular disease, even when traditional variables are taken into account [3].

For clinical management and risk stratification, psoriasis severity is commonly classified based on the body surface area (BSA) involved. Mild psoriasis is defined as affecting up to 3% of the BSA. Moderate disease involves 3% to 10% of the BSA, while severe psoriasis affects more than 10%. This classification is critical, as patients with moderate to severe disease often require systemic therapies or biologics to control the inflammation and are the population most frequently associated with an elevated cardiovascular risk [1].

Psoriasis is a systemic inflammatory disease fundamentally driven by the IL-23/Th17/IL-17 axis. In this pathway, IL-23 from dendritic cells and macrophages promotes Th17 lymphocyte survival, stabilizing the release of proinflammatory cytokines like IL-17A, IL-17F, and IL-22 [3,7-11]. This cascade causes the classic cutaneous inflammation seen in psoriasis via keratinocyte activation, epidermal hyperproliferation, and neutrophil recruitment. Yet the consequences are not merely skin-deep. IL-17A and other cytokines also provoke endothelial dysfunction and vascular activation, directly linking psoriasis to atherosclerosis and cardiovascular risk [12]. This systemic inflammatory profile is amplified because IL-17A is also produced outside of skin plaques by various cells, including dendritic, natural killer (NK), and γδ-T cells. Insight into this mechanism has been key to modern therapeutics [4]. Biologic therapies targeting IL-23 and IL-17 improve skin symptoms and may also reduce vascular inflammation and cardiovascular events, directly addressing the systemic inflammation that harms the cardiovascular system [5,12].

In psoriasis, the body's multisystem inflammatory state is what directly fuels cardiovascular disease. A set of shared mechanisms explains this link. Cytokines from the Th17 pathway, such as IL-17A, team up with classical mediators like TNF-α and IL-6 to launch a vascular inflammatory cascade, all while activating keratinocytes in the skin. This vascular inflammatory cascade leads to endothelial dysfunction and atherosclerosis through the higher production of adhesion molecules and the release of reactive oxygen species (ROS) [4,12]. IL-17A has a key role stimulating endothelial cells to produce intercellular adhesion molecule 1 (ICAM-1), chemokine (C-C motif) ligand 2 (CCL2), and TNF-α and inducing oxidative stress, which facilitates leukocyte adhesion and vascular injury. On top of this, frequent platelet activation in psoriasis worsens endothelial damage by creating neutrophil extracellular traps and amplifying the entire inflammatory process [13,14].

The concept of a "psoriatic march" helps explain how psoriasis evolves from a skin disease into a systemic one, primarily affecting the cardiovascular system. It describes a progression where severe, chronic inflammation triggers early endothelial injury [4]. The march is driven by several factors. One is a clear disturbance in lipid metabolism; patients often exhibit atherogenic dyslipidemia, with elevated levels of oxidized low-density lipoprotein (LDL) and altered lipoproteins that build atheromatous plaque in the vascular intima [13,14]. Another factor is the disease's distinct inflammatory footprint. Specific markers like lipocalin-2, beta-defensin-2, and several interleukins are elevated. Crucially, common indicators such as C-reactive protein (CRP) and erythrocyte sedimentation rate (ESR) do more than just rise; they directly correlate with the severity of the disease.

**Figure 1:**
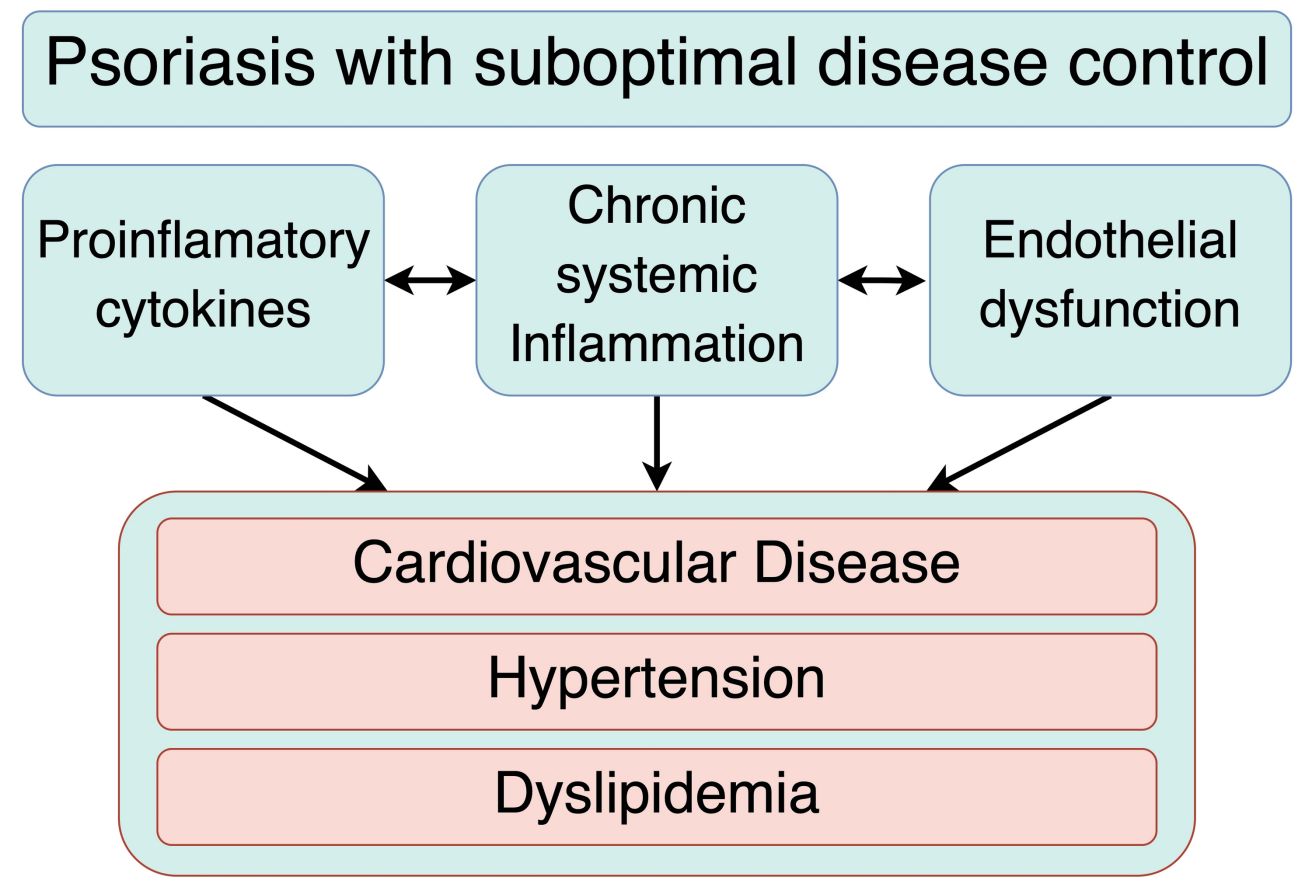
Overview of the Psoriatic March and Its Systemic Consequences Figure created by Dr. Gonzalez-Rodriguez using Draw.io

This confirms that the inflammation affects distant tissues and organs. The outcome of this march from cutaneous to systemic immune activation is an increased risk for many comorbidities: type 2 diabetes, obesity, hypertension, dyslipidemia, and cardiovascular disease (Figure 1). This body of evidence leads to an unavoidable conclusion: psoriasis must be treated as an independent cardiovascular risk factor. The underlying pathophysiology is one of vascular injury, consistently promoted by the body's ongoing immune activation. This activation triggers endothelial dysfunction and a cascade of pathological changes throughout the cardiovascular system, starting early in the disease's course [4,14,15].

The cardiovascular consequences of psoriasis can be severe and often happen prematurely. For example, patients with moderate to severe forms of the disease may suffer a myocardial infarction up to five years earlier than individuals without this condition. This elevated risk, which can reach as high as 50% for overall cardiovascular disease, is largely explained by a greater burden of atherosclerosis. Researchers are able to visualize this burden by using computed tomography (CT) to assess coronary artery calcification (CAC), and studies consistently find that its prevalence and severity are higher in psoriasis patients. However, the vascular damage is not limited to atherosclerosis. Even without significant stenosis, many of these patients also show altered coronary microvascular function, a condition that disrupts blood flow, reduces myocardial perfusion, and further worsens their cardiovascular risk [4,13].

Epidemiological association between psoriasis and major cardiovascular events

A significant amount of epidemiological evidence establishes a link between psoriasis and cardiovascular disease. The risk is most pronounced in moderate to severe cases. Patients may face up to a 50% higher risk of developing cardiovascular disease, which translates to a higher prevalence of acute myocardial infarction and increased cardiovascular mortality [16,17]. This is compounded by significant metabolic disturbances; for example, the functional efficiency of high-density lipoprotein (HDL) lipoproteins can be reduced by roughly 80%, with up to 15% of patients showing suboptimal HDL levels [16].

Specific inflammatory pathways also contribute directly. Elevated IL-17A levels, for instance, are linked to a higher risk of severe and treatment-resistant hypertension. This helps explain why a broad spectrum of adverse outcomes, including myocardial infarction, stroke, thromboembolism, and arrhythmias, are more common in the psoriatic population. As a group, these findings solidify the status of psoriasis as an independent risk factor for cardiovascular events. This recognition makes accurate, individualized cardiovascular risk assessment a critical component in the management of every patient with the disease [16,18].

Cardiovascular risk assessment in patients with psoriasis

Accurate evaluation of cardiovascular risk in people diagnosed with psoriasis is necessary for effective clinical management. Psoriasis involves specific pathophysiological mechanisms that traditional risk estimation tools, such as the Framingham and SCORE algorithms, may not fully account for. This often results in an underestimation of actual cardiovascular risk. As mentioned before, recent studies indicate that patients with psoriasis, especially with a moderate to severe form of the disease, are significantly more likely to experience myocardial infarction at a younger age, with a notable difference of up to five years when compared to people without psoriasis [17,19].

In moderate to severe psoriasis, especially among those undergoing systemic therapy, conventional risk models substantially underestimate cardiovascular risk. These patients show a cumulative incidence of approximately 6.2% for coronary or cerebrovascular events. The age at diagnosis and the total duration of the disease both correlate with sustained vascular inflammation. This suggests that cardiovascular risk may increase progressively the longer a patient lives with psoriasis [16].

For patients on systemic therapy or phototherapy a more aggressive cardiovascular risk strategy is needed. Clinicians should enhance risk estimates, typically by multiplying the calculated score by 1.5. This has to be complemented by proactive screening and monitoring for key comorbidities like hypertension, dyslipidemia, and diabetes mellitus. For patients with an estimated risk of 5% to 7.5% for atherosclerotic cardiovascular disease, initiating moderate-intensity statin therapy is indicated [20,21].

Psoriasis is a systemic disease with a well-documented link to elevated cardiovascular risk. For these reasons, managing patients requires an effective multidisciplinary approach. Coordinating efforts among dermatologists, internists, cardiologists, and other healthcare specialists allows for a more comprehensive evaluation and management of both skin involvement and cardiovascular risks. In addition to pharmacological interventions, educating patients is essential for promoting lifestyle modifications to mitigate harmful habits, including changes such as smoking cessation, increased physical activity, and moderating alcohol consumption. Equally important is the management of body weight, blood pressure, glucose levels, and lipid profiles. This collaborative approach not only facilitates the early detection of comorbidities but also aims to improve long-term outcomes related to both dermatological health and cardiovascular well-being [21,22].

Psoriasis treatment and its impact on cardiovascular health

The primary goals in the clinical management of psoriasis are to reduce inflammatory activity, alleviate cutaneous manifestations, prevent relapses, and improve the overall quality of life for patients. The selection of therapeutic interventions is carefully tailored to the clinical pattern and severity of the disease, with the intensity of treatment proportional to the disease severity. Due to the chronic nature of psoriasis, a long-term, stepwise therapeutic approach is recommended [23].

The choice of appropriate therapy should consider disease severity, stability, location, and patient comorbidities. In mild cases, defined as involvement of up to 3% of body surface area, first-line treatment includes topical agents such as corticosteroids, vitamin D analogs, retinoids, calcineurin inhibitors, keratolytics, and localized phototherapy. Moderate psoriasis involves 3% to 10% of the body surface, while severe disease affects more than 10%. For moderate to severe cases, systemic medications are indicated and may also be used in patients with localized disease who have not responded adequately to topical therapy. Classic systemic agents include methotrexate, acitretin, and cyclosporine. Additionally, biologic therapies that target specific inflammatory pathways have shown significant efficacy. These include TNF-α inhibitors (etanercept, adalimumab, infliximab), IL-17 inhibitors (secukinumab, ixekizumab), and IL-23 inhibitors (ustekinumab, guselkumab) (Table 1) [23-26].

**Table 1:** Psoriasis Severity Classification Based on Body Surface Area and Recommended Therapeutic Options. TNF-α: Tumor Necrosis Factor Alpha; IL: Interleukin. This table was created by Dr. Cristofori with information taken from [23-26].

Disease severity	Body surface area involved	Recommended treatment options
Mild	<3%	Topical therapies (e.g., corticosteroids, vitamin D analogs, retinoids, calcineurin inhibitors, keratolytics) Localized phototherapy
Moderate	3%-10%	Systemic therapies (methotrexate, cyclosporine, acitretin, apremilast) Biologic therapies (TNF-α inhibitors, IL-17 inhibitors, IL-23 inhibitors)
Severe	>10%	Biologic therapies (TNF-α inhibitors, IL-17 inhibitors, IL-23 inhibitors)

Topical treatments for psoriasis

Topical corticosteroids remain among the most effective and safe treatment options for managing psoriasis, especially when lesions are limited to specific body areas. These agents provide several benefits by actively reducing inflammation, suppressing abnormal keratinocyte growth, regulating the immune response, and inducing local vasoconstriction. Corticosteroids work by binding to intracellular receptors that control gene expression and decrease the production of various cytokines and pro-inflammatory mediators. These agents are classified by vasoconstrictive strength into seven classes of potency, from very high (Class 1) to low (Classes 6 and 7). This classification is key to personalizing treatment plans according to the lesion's location, type, and the patient's skin tolerance [24-27].

Topical vitamin D analogs can be used either in combination with corticosteroids or as monotherapy. These agents bind to vitamin D receptors expressed in both keratinocytes and T cells, inhibiting excessive epidermal cell proliferation while promoting proper cellular differentiation. However, their use should be limited in patients with renal insufficiency due to potential adverse effects [25,27,28].

Topical calcineurin inhibitors, which include tacrolimus and pimecrolimus, work by blocking T-cell activation through the suppression of IL-2 and interferon-gamma (IFN-γ) synthesis. These pharmacological agents are primarily used on sensitive or atrophy-prone areas, such as the face and intertriginous zones, because they do not cause skin thinning, a common adverse effect associated with prolonged corticosteroid use [25,29].

Topical keratolytic agents

Topical keratolytic therapies include a range of agents, such as tazarotene and salicylic acid. Tazarotene, a topical retinoid, has been shown to decrease keratinocyte proliferation and promote the desquamation of excessive scale associated with psoriatic plaques. After just a 12-week course of treatment, the outcomes are notable. As many as 63% of patients achieved at least a 50% improvement in their symptoms within this period. Salicylic acid is another effective agent used primarily to reduce hyperkeratosis. However, it carries a risk of local irritation and is contraindicated for pediatric use because of the potential for systemic absorption [25].

Phototherapy

Medical phototherapy utilizes specific wavelengths of light with established therapeutic effects on psoriasis, enabling customization according to the extent of skin involvement. For extensive disease manifestation, full-body phototherapy is employed, whereas localized lesions may be addressed through targeted phototherapy techniques, such as excimer light therapy. This particular approach utilizes high-intensity ultraviolet B (UV-B) radiation at a wavelength of 308 nm [24,25,30].

Narrowband UV-B (NB-UVB) therapy is preferred over broadband UV-B (BB-UVB) due to superior therapeutic outcomes, longer remission periods, reduced incidence of adverse effects, and a lower risk of cutaneous carcinogenesis. NB-UVB is often combined with systemic retinoids to enhance efficacy and further reduce the risk of skin cancer-related complications. In contrast, psoralen + ultraviolet A (PUVA) therapy involves the use of psoralens, such as methoxsalen, which sensitize the skin prior to ultraviolet A (UV-A) exposure. This treatment modality interferes with the processes of DNA replication. Although oral PUVA has demonstrated enhanced efficacy relative to UV-B in certain patient populations, its clinical use has declined considerably due to the increased long-term risk of cutaneous malignancy associated with repeated exposure [24,25].

Oral treatments for psoriasis

The management of moderate to severe psoriasis in the adult population frequently involves the prescription of oral systemic therapies, notably acitretin, apremilast, cyclosporine, and methotrexate. With the exception of cyclosporine, these pharmacological agents are also indicated for patients with persistent localized lesions despite the appropriate application of topical therapies [25]. Generally, oral therapies exhibit reduced efficacy compared to biologic agents; however, they present a more accessible treatment option for patients who prefer oral administration over injections [24,31,32]. These oral agents may be utilized as monotherapy or in conjunction with biologic treatments [31].

Methotrexate acts by inhibiting the enzyme dihydrofolate reductase, thereby interfering with cellular proliferation and reducing the inflammatory response. It is one of the most widely used systemic agents; however, its use requires regular monitoring due to potential adverse effects, including myelosuppression and hepatotoxicity. Folic acid supplementation is recommended to reduce the incidence of adverse effects such as stomatitis and hematologic toxicity. Moreover, due to its teratogenic potential, methotrexate must be discontinued at least three months prior to conception in both men and women [23,31]. Cyclosporine, on the other hand, suppresses T-cell function and has demonstrated considerable efficacy in addressing acute severe psoriasis flares, with clinical improvements often noticeable within a matter of weeks. However, the long-term application of cyclosporine is constrained by risks related to nephrotoxicity and elevated blood pressure, necessitating limitations on treatment duration. Acitretin, a non-immunosuppressive oral retinoid, is typically reserved for severe forms of psoriasis such as pustular or erythrodermic variants. Its therapeutic impact is characterized by a gradual onset, typically requiring several months to achieve optimal efficacy. Due to its teratogenicity, its use is strictly contraindicated in women of childbearing age. Apremilast provides another therapeutic pathway, working as a selective phosphodiesterase 4 (PDE4) inhibitor to modulate key inflammatory processes. Its efficacy in both plaque psoriasis and psoriatic arthritis makes it a primary choice for patients who do not respond adequately to other systemic therapies. Despite its general tolerability, potential side effects such as nausea, diarrhea, and weight loss must be considered [23,31,32].

Biologic therapies

Biologic agents have transformed contemporary dermatology, offering therapies with both excellent tolerability and long-term efficacy. While they are a cornerstone for severe psoriasis, their use extends to difficult-to-treat areas like the scalp, palms, and nails, and to cases complicated by psoriatic arthritis. These therapies are classified by their mechanism of action [33].

One of the most established classes is the TNF-α inhibitors, such as adalimumab and infliximab. Although effective, they carry a potential risk of reactivating latent infections like hepatitis and tuberculosis. Their onset of action can vary, with infliximab often working within eight to 10 weeks, while others can take up to 16 weeks. These blockers are frequently preferred for patients who also have inflammatory bowel disease. The IL-17 inhibitors, including secukinumab and ixekizumab, are known for their particularly rapid therapeutic effect and exceptional efficacy in challenging forms, such as nail psoriasis. However, they have been associated with mucocutaneous candidiasis and a risk of exacerbating inflammatory bowel disease. More recent developments have focused on increased selectivity. Ustekinumab, which targets both IL-12 and IL-23, has demonstrated strong clinical efficacy. A newer generation of inhibitors targeting only IL-23, such as guselkumab and risankizumab, offers highly specific and sustained action, often with convenient dosing and an excellent safety profile. The growing variety of these biologics illustrates a paradigm shift in dermatology toward highly personalized treatments for complex conditions [24,25,31,34,35].

Impact of psoriasis treatment on cardiovascular health

Timely and effective management of psoriasis, particularly in patients with severe disease, may help reduce the risk of developing associated cardiovascular conditions, as biologic therapies can attenuate the prolonged immunologic activity contributing to cutaneous and vascular injury [6]. 

Research has shown that the use of biologic therapies, particularly TNF-α inhibitors, offers benefits beyond cutaneous control in patients with psoriasis, including a significant reduction in the incidence of cardiovascular events, such as acute myocardial infarction. This cardioprotective effect has been linked to improved coronary perfusion, as evidenced by a significant increase in coronary flow reserve. Imaging studies have also revealed greater stability of subclinical coronary artery disease in patients receiving biologic therapy, in contrast to those not treated with biologics, who tend to show progression of vascular damage. Moreover, TNF-α, IL-17, and IL-23 inhibitors have been associated with favorable effects on endothelial function and reduced oxidative stress, key mechanisms in the prevention of cardiovascular conditions such as atherosclerosis, peripheral artery disease, and cerebrovascular events. An important reduction in inflammatory biomarkers, including CRP and VEGF has also been observed after several weeks of treatment, further supporting the potential cardioprotective role of these therapies [5,6].

Limitations, recommendations, and future perspectives

While growing evidence supports the association between psoriasis and increased cardiovascular risk, further research is needed to fully characterize the long-term systemic impact of this disease and its treatments [5,6,16]. Current cardiovascular risk assessment tools may underestimate the true risk in patients with moderate to severe psoriasis, as they do not incorporate markers of systemic inflammation or account for disease duration and severity [16,19]. Clinicians are encouraged to adopt individualized management strategies, including correction factors in risk estimation, regular screening for metabolic comorbidities, and early initiation of preventive therapies such as statins when indicated [21]. Future studies should prioritize the development and validation of psoriasis-specific cardiovascular risk models. Importantly, randomized controlled trials with long-term follow-up are needed to determine the true cardioprotective effect of systemic and biologic therapies, beyond surrogate endpoints. In addition, research should explore the potential of early therapeutic intervention to modify cardiovascular risk trajectories in high-risk populations. A multidisciplinary, preventive approach that integrates dermatologic care with cardiovascular risk reduction strategies remains essential for improving long-term outcomes in patients with psoriasis.

## Conclusions

Psoriasis, particularly in its moderate to severe forms, is increasingly recognized as a systemic inflammatory condition associated with a significantly elevated cardiovascular risk. This is reflected in the higher prevalence of acute myocardial infarction and cardiovascular mortality observed in affected individuals compared to the general population. Biologic therapies targeting TNF-α, IL-17, and IL-23 have demonstrated not only substantial dermatologic benefits but also a potential role in reducing cardiovascular events by mitigating systemic inflammation.

From a clinical perspective, comprehensive cardiovascular risk assessment is essential in the management of psoriasis, particularly in patients with extensive or long-standing disease. There is a clear need for randomized clinical trials with long-term follow-up to better define the cardiovascular impact of systemic therapies. These findings provide significant evidence supporting the classification of psoriasis as a multisystem inflammatory condition with important vascular implications. They support the adoption of an integrated therapeutic approach that incorporates cardiovascular prevention into routine dermatologic care, shifting the focus from isolated skin lesions to the broader systemic implications of the disease.
